# Single nucleotide polymorphisms of CD20 gene and their relationship with clinical efficacy of R-CHOP in patients with diffuse large B cell lymphoma

**DOI:** 10.1186/1475-2867-13-58

**Published:** 2013-06-10

**Authors:** Huirong Ding, Xuan Jin, Ning Ding, Zhiying Fu, Yuqin Song, Jun Zhu

**Affiliations:** 1 Key laboratory of Carcinogenesis and Translational Research (Ministry of Education), Central Laboratory, Peking University Cancer Hospital & Institute, Beijing 100142, China; 2 Department of Internal Medicine Oncology, Peking University First Hospital, Beijing 100034, China; 3 Key laboratory of Carcinogenesis and Translational Research (Ministry of Education), Department of Lymphoma, Peking University Cancer Hospital & Institute, Beijing 100142, China

## Abstract

**Background:**

R-CHOP has significantly improved survival rates of patients with diffuse large B cell lymphoma (DLBCL) by ~20% as compared to CHOP. CD20 antigen, highly expressed on more than 80% of B-cell lymphomas, is the target for rituximab. The goal of our study was to examine polymorphism in the CD20 gene in Chinese DLBCL population and whether CD20 gene polymorphism is associated with clinical response to R-CHOP.

**Method:**

CD20 gene polymorphism was detected in the entire coding regions including 6 exons by polymerase chain reaction (PCR)-sequencing assay in 164 patients with DLBCL. Among them, 129 patients treated with R-CHOP as frontline therapy (R ≥ 4 cycles) were assessable for the efficacy.

**Results:**

Polymorphisms at three single nucleotides (SNP) were identified in the entire coding regions of the CD20 gene in the 164 patients. One of them, CD20 Exon2 _[216]_ was found to be highly correlated with response to R-CHOP. Patients with homozygous C genotype showed a trend toward higher overall response rate than others with CT plus TT genotype (90.6% vs. 79.5%; *P* =0.166). A trend toward higher complete remission (CR) rate was observed in patients with homozygous C genotype (67.4%) compared with CT plus TT genotype (47.1%) (*P* = 0.091).

**Conclusion:**

These results suggest that there are 3 SNPs in CDS of the CD20 gene in Chinese DLBCL population. The CC genotype at Exon2 _[216]_ appears to be associated with favourable response to R-CHOP.

## Introduction

Genetic polymorphisms are variants in individual genomes and remain constant throughout a person’s lifetime. Many genetic polymorphisms contribute to variability in drug pharmacokinetic and pharmacodynamic processes [1]. The relationship between therapeutic efficacy and gene polymorphism has been extensively studied for a few monoclonal antibodies [2,3].

Rituximab is a chimeric monoclonal antibody targeting the CD20 antigen on normal and neoplastic B cells [4-7]. R-CHOP (rituximab, cyclophosphamide, doxorubicin, vincristine and prednisone) has significantly improved survival rates of patients with diffuse large B cell lymphoma (DLBCL) [8-11]. CD19 and other monoclonal antibodies are also being explored for novel lymphoma therapies [12].

CD20 was first identified as a B-cell specific marker in 1980 [13], which was highly expressed on more than 80% of B-cell lymphomas but not on stem cells, pro-B cells, normal plasma cells, or other normal tissues [14].The CD20 gene, namely MS4A1 gene, is located on chromosome 11q12-q13.1 with 6 exons in its coding sequence (CDS) [15,16]. To date, 509 single-nucleotide polymorphisms (SNPs) have been reported for the CD20 gene. Among them, 57 SNPs are located in CDS, but there were no genotype and allele frequency data for most SNPs. In particular, there has been no report on the relationship between CD20 gene SNPs and their impacts on the response to R-CHOP in DLBCL patients. Several clinical studies offered scanty information about the CD20 gene polymorphism when they researched the CD20 mutations in tumour tissues. Johnson et al. described that the CD20 gene mutations in the rituximab epitope are rare and no SNPs were detected in exon 5 of the CD20 gene [17]. Another study found no mutation in the CDS of the CD20 gene in tumours from 23 patients, with only one case showing a SNP [16].

Therefore, this study examined polymorphism of the CD20 gene in Chinese DLBCL population and the relationship between the polymorphism and clinical efficacy in patients with DLBCL treated with R-CHOP.

## Methods

### Study population

The clinical research protocol had been approved by our Institutional Review Board (IRB). This study had been approved by the Research and Ethical Committee of Peking University School of Oncology. A written informed consent had been obtained from each patient participated in this study.

This study included 164 patients with CD20+ DLBCL confirmed by our Department of Pathology according to the World Health Organization classification. All patients received R-CHOP (120 patients) or R-CHOP-like (44 patients) chemotherapy regimen between June 2007 and December 2010 at Beijing Cancer Hospital, Peking University School of Oncology. For elderly patients or patients with other complications, the dosage of CHOP was changed, which is not standard dosage of CHOP, so called R-CHOP-like. R-CHOP chemotherapy was administered as follows: one course of chemotherapy consisted of an intravenous infusion of Cyclophosphamide 750 mg/m^2^, adriamycin 50 mg/m^2^, vincristine 2 mg, and an oral administration of 100 mg prednisone on days 1 to 5, which was repeated every 3 weeks. Rituximab 375 mg/m^2^ was infused over 4 to 6 hours on day 1 before CHOP or CHOP-like chemotherapy was started. Among the 164 patients, 129 received frontline R-CHOP and were evaluable for clinical efficacy. Of the 129 patients, 31 patients received involved-field radiation. The response to R-CHOP therapy was evaluated after completion of 2 to 3 courses of therapy and 1 to 2 months after completion of all therapy plans, then every 3 months for the first year and every 6 months thereafter until progression.

### Analysis of the CD20 gene polymorphisms

Blood samples were obtained from all lymphoma patients before the initiation of therapy for genetic analysis. Genomic DNA was prepared from peripheral-blood mononuclear cells using Blood Genomic DNA extraction kit following the manufacturer’s instructions (Bioteke Corporation, China). All 6 exons of the CD20 gene were examined by polymerase chain reaction (PCR)-sequencing assay. Genomic DNA (30 ng per 30 μl reaction) was amplified with GoTaq DNA polymerase (Promega Cor., WI, USA) and exon-specific primer sets for each of the 6 exons [12]. PCR reaction conditions were programmed on a thermo cycler (Gene Cycler TM, Bio-Rad, CA, USA) as follows: denaturation at 94°C for 5 min, followed by 35 cycles of 94°C for 30s, 56°C for 30 s, 72°C for 45 s. The last cycle was followed by 72°C for 7 min. Amplified products were analyzed by gel electrophoresis on 2% agarose gels. All fragments were purified with the AxyPrep DNA Gel Extraction kit according to the manufacturer’s instructions (Axygen Sci. Inc., CA, USA). Those purified products were sequenced using an ABI 3730XL Avant Genetic Analyzer (Applied Biosystems Inc., CA, USA). Finally, the sequences were analyzed with the software Seqman (DNASTAR Inc., WI, USA).

### Definitions

The patients who had heterozygous (CT) or homozygous T (TT) genotype of CD20 Exon2_[216]_ were designated as T carriers. Clinical responses were scored with the criteria formulated by International Working Group [18] and were determined by physical examination and confirmed by computed tomography or ultrasound. The latter was only used for evaluating superficial lymph nodes. Disease specific survival (DSS) was calculated as the day 1 of the first cycle of R-CHOP until death for DLBCL, or until the last follow-up available. The progression-free survival (PFS) was calculated from day 1 of the first cycle of R-CHOP until disease progression, or death for any cause. If a patient had not progressed or died, PFS was censored at the time of last follow-up.

### Statistical analysis

The clinical characteristics and response rate of the patients were compared using χ^2^ test and Fisher’s Exact Test according to the CD20 SNP. The association of polymorphism with DSS and PFS was analyzed using Kaplan-Meier curves and the log-rank test. The prognostic factors were evaluated by the Cox regression model. Differences between groups were regarded as significant with a *P* < 0.05. All tests of statistical significance were two-sided. The analysis was performed using the SPSS16.0.

## Results

### Patient characteristics

The general features of the patients in this study are summarized in Table 1, including 81 female and 83 male. The median age at diagnosis was 53 years (range, 15–90 years). Eighty nine (54%) patients were in stages 3 or 4 and 50 (30%) patients had intermediate-to-high or high International Prognostic Index (IPI) scores. Bone marrow was involved by lymphoma in 6 patients (4%) at diagnosis. R-CHOP followed by involved-field radiation was given to 31 (19%) patients. R-CHOP as a front-line regimen was administrated to 129 patients whose clinical efficacy was evaluable for this study. A median of 6 rituximab doses were given (range, 4–14), and a median of 6 cycles of chemotherapy was given (range, 2–8 cycles).

**Table 1:** Patient’s characteristics and their correlations with CD20 Exon2_[216]_ genotype

**Clinical parameters**	**No.**	**Genotype**		P*	**Clinical parameters**	**No.**	**Genotype**		P*
		**CC**	**CTplusTT**				**CC**	**CTplusTT**	
Gender					Bulky mass				
Male	83	63	20	0.787	≥10cm	18	11	7	0.249
Female	81	60	21		<10cm	146	112	34	
Age					Localized				
≤60	102	76	26	0.852	Yes	25	19	6	0.900
>60	62	47	15		No	139	104	35	
B symptoms					No Extra Nodal				
Positive	62	46	16	0.852	≤1	122	90	32	0.535
Negative	102	77	25		>1	42	33	9	
LDH					Incidence site				
Positive	77	55	22	0.320	Lymph node	93	68	25	0.524
Negative	87	68	19		Extralymph	71	55	16	
β_2_-MG					IPI				
Positive	49	35	14	0.684	0-2	114	84	30	0.557
Negative	106	79	27		3-5	50	39	11	
Stage					Molecular subtypes				
I-II	75	55	20	0.651	GCB	28	21	7	0.868
III-IV	89	68	21		Non-GCB	113	83	30	

Abbreviations: *LDH* lactate dehydrogenase level, *MG* macroglobulin, *IPI* international prognostic index, *GCB* germinal centre B cell, *χ^2^ test.

### CD20 gene polymorphism

Three SNPs were identified in the entire coding regions (6 exons) of the CD20 gene in this DLBCL patient population (Table 2). All three SNPs were located in exon1 and exon2. Exon 3–6 showed no SNPs. The c.111G > C in exon 1, the c.208C > T and c.216C > T (CD20 Exon2_[216]_) in exon2 have been previously reported separately as rs200805059, rs79703274 and rs2070770 (http://www.ncbi.nlm.nih.gov/SNP/snp_ref.cgi?locusId=931). Genotype and allele frequencies of the CD20 gene polymorphism in 164 patients with DLBCL were analyzed (Table 3). The frequency of the CD20 Exon2 _[216]_ C allele among the 164 patients was 0.869, whereas the frequency of the CD20 Exon2_[216]_ T allele was 0.131. Seventy-five percent (123 of 164) of patients were homozygous for CD20 Exon2_[216]_ C allele, 23.8% (39 of 164) were heterozygous (C/T), and 1.2% (2 of 164) were homozygous for CD20 Exon2_[216]_ T allele (Table 3). The genotype distribution of DLBCL population enrolled in this study was in Hardy-Weinberg equilibrium with regard to the CD20 Exon2_[216]_ polymorphism examined (*P* = 0.57).

**Table 2:** CD20 polymorphisms in 164 Chinese people with DLBCL

Nucleotide change*	**Effect on protein**	**location**	**Ref**
c.111G > C	p.L37L	Exon1	rs200805059
c.208C > T	p.L70L	Exon2	rs79703274
c.216C > T	p.I72I	Exon2	rs2070770

Abbreviations: *G* Guanine, *C* Cytosine, *T* Thymine, *L* Leu, leucine, *I:Ile* isoleucine.

* Position in CD20 cDNA is according to Reference Sequence: NM_021950.3. The mutation nomenelature follows the instructions provided by the HGVS (http://www.hgvs.org). The DNA polymorphisms numbering is based on cDNA sequence with +1 corresponding to the A of the ATG translation initiation codon.

**Table 3:** Genotype and allele frequencies of CD20 polymorphisms in 164 Chinese patients with DLBCL

**SNP**	**Genotype frequencies**										**Allele frequencies**						
	**Genotype**	**freq**	**count**	**Genotype**	**freq**	**count**	**Genotype**	**freq**	**count**	**Total**	**Allele**	**freq**	**count**	**Allele**	**freq**	**count**	**Total**
c.111G>C	GG	0.988	162	GC	0.012	2	CC	0	0	164	G	0.994	326	C	0.006	2	328
c.208C>T	CC	0.994	163	CT	0.006	1	TT	0	0	164	C	0.997	327	T	0.003	1	328
c.216C>T	CC	0.750	123	CT	0.238	39	TT	0.012	2	164	C	0.869	285	T	0.131	43	328

### Patient characteristics according to Exon2_[216]_ allele status

There was no significant difference in patients’ disease features between the CD20 Exon2_[216]_ CC and CT plus TT polymorphism groups (Table 1).

### Clinical responses and Exon2 _[216]_ Polymorphism

Although not statistically significant, the patients with homozygous C genotype showed a trend toward higher overall response rate than those with CT plus TT genotype (90.6% vs. 79.5%; *P* =0.166, Table 4). The trend seems to be largely due to a better complete response rate (CR). Higher CR rate was observed in patients with homozygous C genotype (67.4%) compared to those with CT plus TT genotype (47.1%) (*P* = 0.091). When the rate of CR was compared with that of non-CR (PR + SD + PD), the difference in CR rate was significant in favour of the homozygous C genotype (χ^2^, 4.384; *P* = 0.036) (Table 4). When the patients were subdivided into GCB and non-GCB lymphoma groups, the better response rate was observed in non-GCB groups (59.0% vs. 37.5%; *P* =0.074), not in GCB (80.0% vs. 71.4%; *P* =0.633).

**Table 4:** Clinical response to R-CHOP therapy according to CD20 Exon2_[216]_ Polymorphism

** Response**	**CD20 exon2**_**[216]**_**genotype n (%)**		*P *Value*
	**CC No.(%)**	**CT + TT No.(%)**	
No.(N = 129)	95	34	
CR	64(67.4)	16(47.1)	0.091
PR	22(23.2)	11(32.4)	
SD/PD	9(9.4)	7(20.5)	
ORR(CR + PR)	86(90.6)	27(79.5)	0.166^#**1**^
CR	64(67.4)	16(47.1)	0.036^#**2**^
Non-CR	31(32.6)	18(52.9)	

Abbreviations: *CR* complete response, *PR* partial response, *SD* stable disease, *PD* progressive disease, *ORR* (OR + PR), overall response, non-CR, PR + SD + PD.

#1 P = 0.166 when comparing ORR with SD/PD;#2 P = 0.036 when comparing CR with non-CR (PR + SD + PD) ;* χ^2^ test.

### Survival analysis according to CD20 Exon2_[216]_ genotype

**Figure 1:**
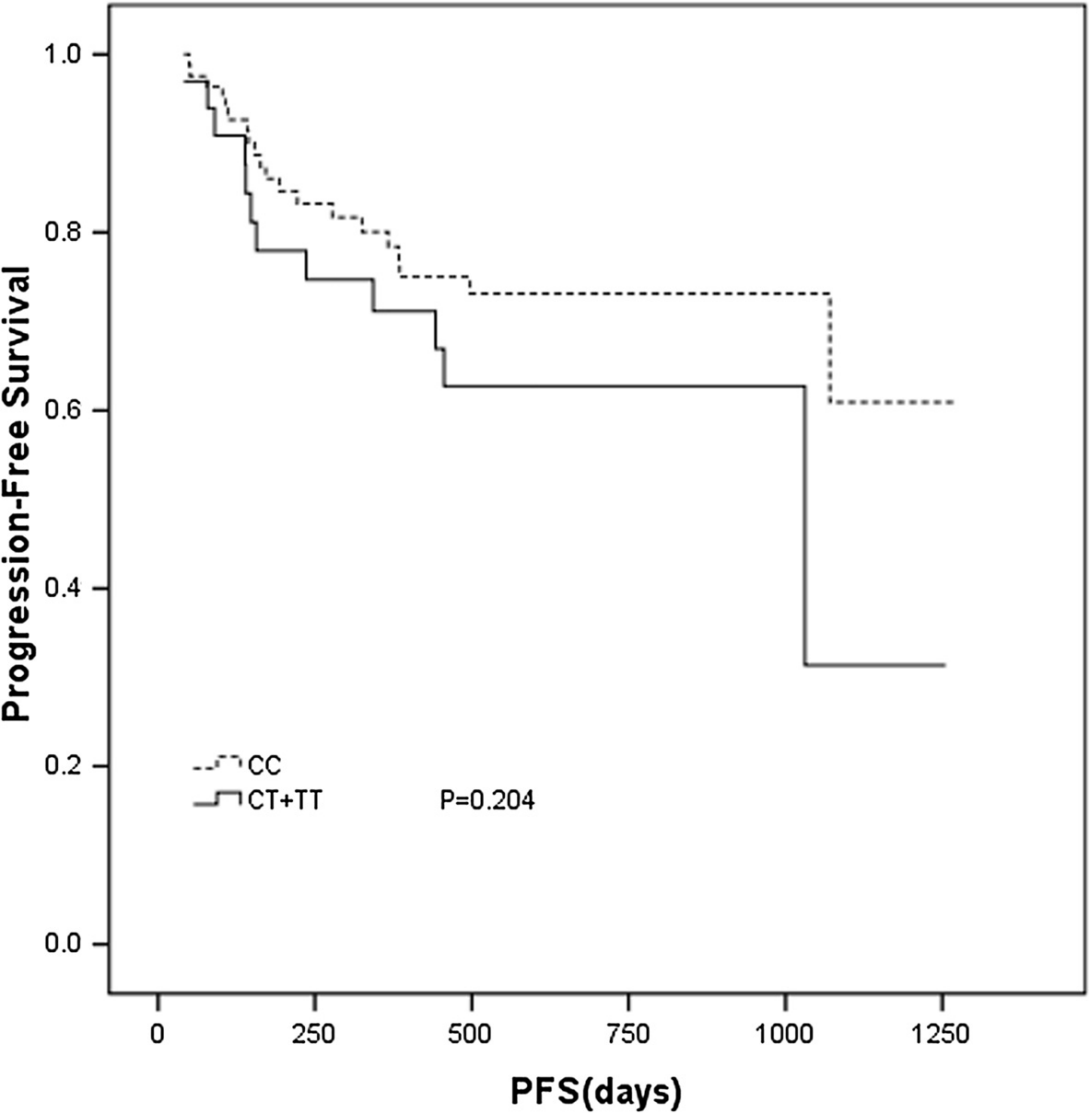
**Progression-free survival (PFS) in DLBCL subjects with CD20 Exon2_[216]_ polymorphism.** Kaplan-Meier curve of PFS was plotted by CD20 Exon2 _[216]_ CC and CT plus TT genotype.

**Figure 2:**
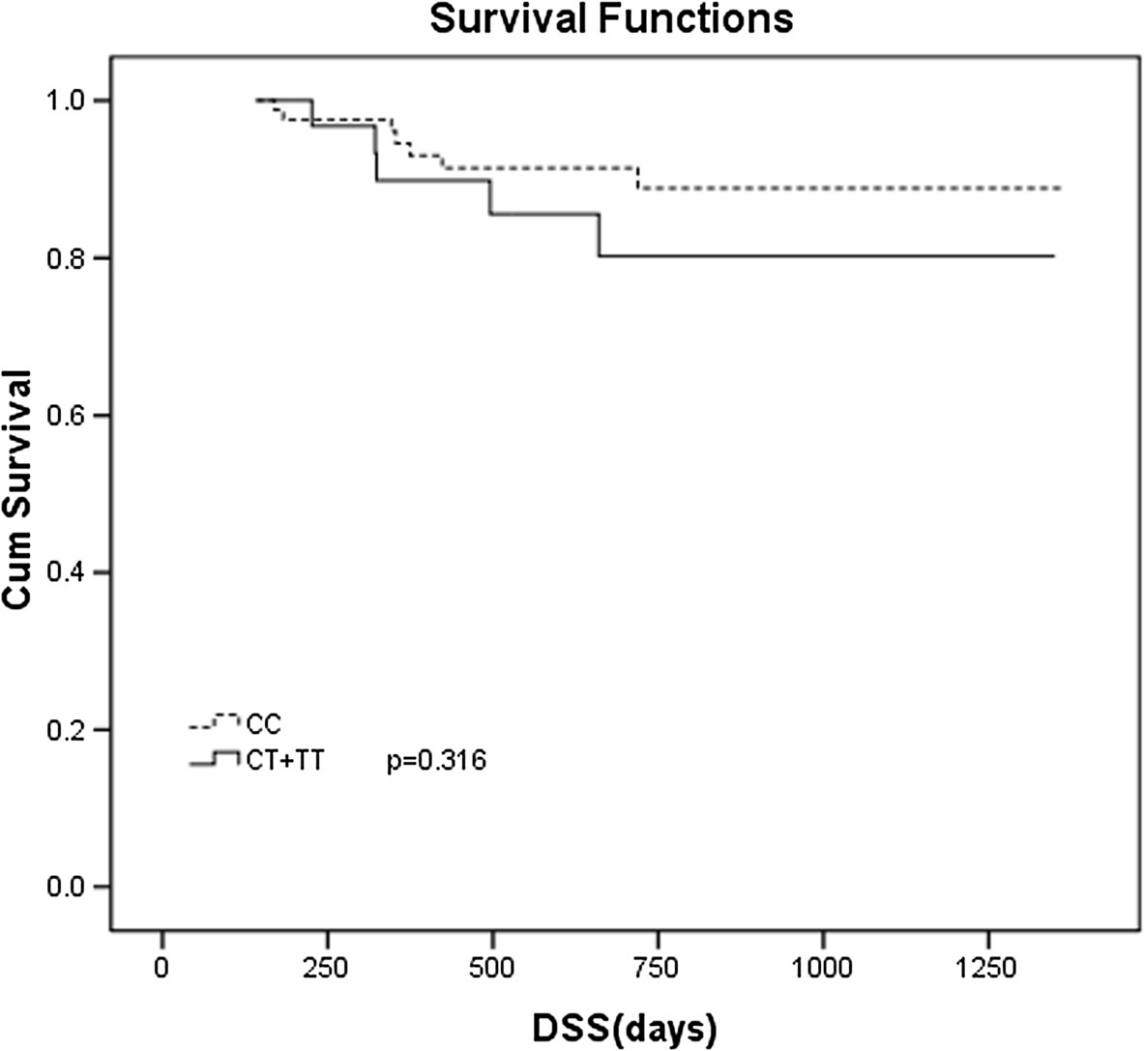
**Disease specific survival (DSS) in DLBCL subjects with CD20 Exon2_[216]_ polymorphism.** Kaplan-Meier curve of DSS was plotted by CD20 Exon2 _[216]_ CC and CT plus TT genotype.

After a median follow-up time of 524 days (range, 60–2073 days), 32 (25%) patients relapsed or progressed, and 18 (14%) died. Seven patients participated in a clinical trial evaluating everolimus (RAD001) and were censored for PFS analysis. The patients with homozygous C genotype had a median PFS of 465 days (range, 50–1275 days) versus 442 days (range, 41–1255 days) for the rests, but the difference did not reach statistical significance (*P* = 0.204) (Figure 1). Survival data was available for 123 patients. Six patients were censored for not being died of DLBCL. The DSS was 536 days (range, 143–1364 days) for homozygous C patients, and 571 days (range, 142–1350 days) for T carriers (*P* = 0.316, Figure 2). This difference was neither statistically significant between GCB and non-GCB lymphoma groups (data not shown).

### Multivariate analysis

A multivariate analysis was done to evaluate the following variables on DSS and PFS: age (≤60 vs. >60 years), stage (stages 1,2 vs. 3,4), B symptoms (positive vs. negative), β_2_-microglobulin level (normal vs. abnormal), LDH (normal vs. abnormal), IPI score (0–2 vs. 3–5), No. of extra nodal sites (≤1 site vs. ≥2 sites), bulky mass (≥10 cm vs. <10 cm), and Exon2_[216]_ (homozygous C vs. T carriers). This analysis confirmed that LDH (*P* = 0.034, HR 4.23, 95% CI 1.113-16.057) was poor prognostic factor.

## Discussion

The focus of several previous study is on relationship of mutations or/and polymorphisms of CD20 gene with the response to R-CHOP in diffuse large B cell lymphoma patients. However, in Sar’ study, the sample number is less. Specimens are from 23 patients diagnosed with DLBCL and treated with R-CHOP were included in the study [16]. In Johnson’ study, only mutations or/and polymorphisms in exon5 of CD20 gene were determined [17]. In our study, all 6 exons of the CD20 gene were examined. Specimens are from 164 patients diagnosed with DLBCL and treated with R-CHOP were included in our study. This study described polymorphism in CD20 exons in Chinese DLBCL patient population for the first time. There totally are 3 SNPs in CDS of the CD20 gene in this population (Table 2). For population diversity, there are still no genotype and allele frequency data about the c.111G > C (rs200805059) and the c.208C > T (rs79703274) in the NCBI SNP database prior to this study. Here we obtained the genotype frequencies and allele frequencies for above two SNPs in Chinese DLBCL patient population (Table 3). C allele of c.111G > C and T allele of c.208C > T are rare in this population. For the c.216C > T (CD20 Exon2_[216]_, rs2070770 ) the genotype and allele distribution in DLBCL population from NCBI SNP database was similar to those obtained from Han Chinese in Beijing, China (Table 3). Thus, the overall allelic distribution in Chinese DLBCL patients was not statistically different from the allelic distribution in other populations. In addition, the DLBCL population enrolled in this study was in Hardy-Weinberg equilibrium with regard to the CD20 Exon2_[216]_. More importantly, the results described above provide first evidence that the genetic polymorphism, CD20 Exon2_[216]_ may have an impact on clinical efficacy of R-CHOP (Table 4).

Several possible explanations for our findings can be provided. CD20 is highly expressed on more than 80% of the B-cell lymphomas [19]. The previous study suggested deletion mutations of CD20 gene changed the expression level of CD20 antigen, which may be related to the resistance after rituximab therapy [20]. Therefore the CD20 Exon2_[216]_ may potentially alter the level of CD20 antigen expression, thus affecting the clinical response to R-CHOP. We have used immunohistochemical method (IHC) to detect correlation of CD20 expression intensity with CD20 Exon2 _[216]_, no significant difference was found (Data was not shown). The CD20 Exon2_[216]_ C for T substitution is a synonymous SNP of the third base of the codon for Ile72. Because synonymous SNPs encode a change in the DNA sequence without altering the resultant protein sequence, such “silent” changes were long assumed to be inconsequential. However, there is clear and accumulating evidence from recent work that synonymous SNPs can alter the expression, conformation, or function of a protein by a variety of mechanisms, including altering the efficiency of gene translation, affecting the stability of mRNAs and regulating the splicing process of mRNAs [21]. For example, a synonymous SNP, the C3435T polymorphism in the Multidrug Resistance 1(MDR1) gene affects MDR1 gene product pP-glycoprotein (P-gp) activity through altering its conformation [18,22]. Synonymous SNPs within the DRD2 transcript can reduce the stability of the mRNA and thus the expression of the dopamine receptor [23]. Synonymous SNPs in CHRNA4 alter the receptor response to Ach [24]. To date, silent SNPs have been reported in association with more than 40 diseases that have genetic bases [25]. Meanwhile, according to biased codon usage, synonymous SNP substituting a rare codon for a common codon encoding the same amino acid may directly impact the translation kinetics of a protein, resulting in its function alteration [22,26]. In the case of CD20 Exon2_[216]_, the ATT > ATC transversion represents a change from a rare (ATT, 13.0 per thousand) codon encoding isoleucine to a more frequently used codon (ATC, 29.9 per thousand; frequencies obtained from the Codon Usage Database:http://www.kazusa.or.jp/codon/). Alternatively, CD20 Exon2_[216]_ may be commonly inherited as part of a haplotype and exist in linkage disequilibrium with other disease-associated molecular markers [20], such as SNPs from a key gene involved in ADCC or cell-dependent cellular cytotoxicity (CDCC).

While we explored a correlation between clinical outcome and CD20 Exon2 _[216]_, we found there are no statistically significant correlation between CD20 Exon2_[216]_ genotypes and PFS and DSS (Figures 1 and 2). In addition, the CD20 Exon2_[216]_ was not an independent predictor for prognosis. The possible explanation is that as a target, CD20 antigen is an important factor for initial response, but not for duration of R-CHOP response in DLBCL patients. The latter has different mechanisms and may be more dependent on immunity status of DLBCL patients. Meanwhile, patients with DLBCL, GCB lymphoma have better survival than non-GCB lymphoma. In our study, the difference of the overall survival between homozygous C patients, and T carriers did not reach statistical significance. This difference was neither statistically significant between GCB and non-GCB lymphoma groups in subgroup analysis (data not shown). Maybe it is a prognostic factor independent of molecular subtypes (GCB or non- GCB). Or it is possible that this may be due to the small number of patients that preclude reliable analysis.

## Conclusion

In conclusion, this study points to a possible association between polymorphism in the CD20 gene and the response to R-CHOP in DLBCL patients. Ongoing studies will explore the impact of this SNP in a larger population of DLBCL and verify it with laboratory experiment.

## Competing interests

The authors declare that they have no competing interests.

## Authors’ contributions

ZJ and SYQ designed the study and reviewed the final manuscript. DHR and JX performed and evaluated the experiments. DN helped to perform the experiments. FZY helped to collect the samples. JX collected and analyzed data. JX, SYQ and DHR wrote the manuscript. DHR and JX contributed equally to this work. All authors read and approved the final manuscript.
